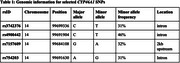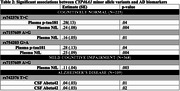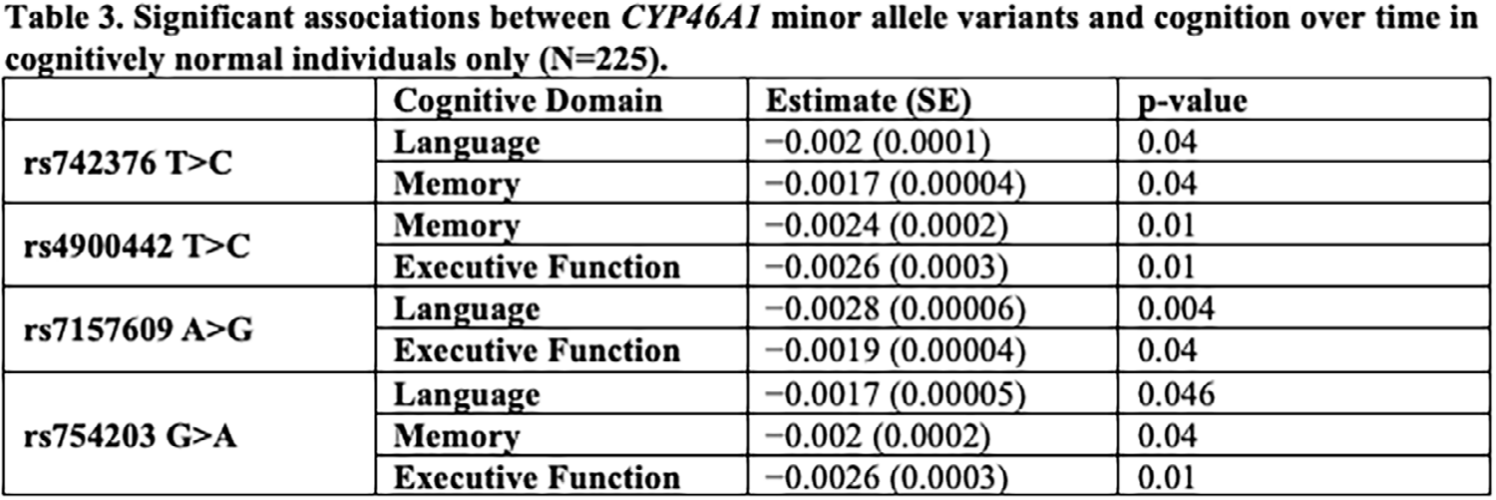# Brain cholesterol metabolism in Alzheimer's disease: Insights from ADNI and the UK BioBank

**DOI:** 10.1002/alz70855_105070

**Published:** 2025-12-24

**Authors:** Myuri Ruthirakuhan, Yuen Yan Wong, Sofia Perfetto, Lisa Y. Xiong, Che‐Yuan Wu, Si Won Ryoo, Daniel K Mori‐Fegan, Meghan J. Chenoweth, Walter Swardfager

**Affiliations:** ^1^ University of Toronto, Toronto, ON, Canada; ^2^ Hurvitz Brain Sciences Program, Sunnybrook Research Institute, Toronto, ON, Canada; ^3^ Sandra Black Centre for Brain Resilience and Recovery, Sunnybrook Research Institute, Toronto, ON, Canada; ^4^ Sunnybrook Research Institute, Toronto, ON, Canada; ^5^ Campbell Family Mental Health Research Institute, Centre for Addiction and Mental Health, Toronto, ON, Canada; ^6^ Dr. Sandra E. Black Centre for Brain Resilience and Recovery, Toronto, ON, Canada

## Abstract

**Background:**

Brain cholesterol metabolism plays a critical role in maintaining neuronal health and cognitive function. The *CYP46A1* gene encodes cholesterol 24‐hydroxylase, which converts brain cholesterol to 24S‐hydroxycholesterol (24S‐HOC), a metabolite that can pass the blood brain barrier. In Alzheimer's disease (AD) and vascular dementia, minor allele variants of *CYP46A1*, including rs3742376‐C>T, rs4900442‐C>T, rs7157609‐G>A, and rs754203‐A>G, have been associated with increased peripheral blood 24S‐HOC concentrations, indicative of impaired brain cholesterol metabolism. However, it is unclear how brain cholesterol metabolism is associated with AD‐specific markers, cognitive function and dementia risk.

**Method:**

Using the Alzheimer's Disease Neuroimaging Initiative (ADNI), and UK Biobank (UKB) datasets, we conducted a candidate gene study with the four *CYP46A1* variants previously associated with increased 24S‐HOC production (Table 1). In ADNI, linear regressions were used to investigate the association between the *CYP46A1* variants and AD biofluid markers (amyloid‐beta, phospho‐tau, neurofilament light [NfL], and glial fibrillary acid protein). Linear mixed models were used to investigate the association between *CYP46A1* variants and cognition (language, memory, executive function, and attention). All analyses were stratified by cognitively normal (CN), mild cognitive impairment, and AD, and adjusted for age, sex, MMSE, and apolipoprotein (APOE) ε4 allele status. In UKB, Cox proportional hazards models were used to investigate the association between *CYP46A1* variants and dementia risk in participants dementia‐free at baseline (age≥55). All analyses were performed using additive genetic models. Covariates included age, sex, APOEε4 allele status, hypertension, dyslipidemia, smoking history, obesity, and diabetes.

**Result:**

In ADNI (*N* = 702), *CYP46A1* minor allele variants were significantly associated with lower amyloid‐beta, higher *p*‐tau, and higher NfL (Table 2). In CN individuals, these variants were also associated with poorer cognition in language, memory, and executive function (Table 3). In UKB (*N* = 26,620), the minor C allele of rs4900442 was associated with increased 15‐year dementia risk [HR(95% CI): 1.17 (1.02‐1.34), *p* = .03] compared to non‐carriers.

**Conclusion:**

These findings implicate brain cholesterol metabolism in cognitive decline and dementia risk, with specific effects on AD pathophysiology. Brain cholesterol metabolism is implicated as a potential pathway for targeted preventative and therapeutic intervention strategies.